# Practical applications of CISS MRI in spine imaging

**DOI:** 10.1016/j.ejro.2019.06.001

**Published:** 2019-06-27

**Authors:** Zhixi Li, Yingming Amy Chen, Daniel Chow, Jason Talbott, Christine Glastonbury, Vinil Shah

**Affiliations:** Department of Radiology and Biomedical Imaging, University of California, 505 Parnassus Avenue, M-391, San Francisco, CA 94143-0628, USA

**Keywords:** Constructive interference in steady state (CISS), Magnetic resonance imaging, Fast imaging employing steady state acquisition (FIESTA), Spine imaging, Balanced steady state free precession (b-SSFP)

## Abstract

Routine magnetic resonance imaging evaluation of the spine is often limited by low spatial resolution and artifacts resulting from cerebrospinal fluid pulsation. Balanced steady-state free precession sequences can supplement routine spin echo sequences and provide exquisite anatomic detail and high cerebrospinal fluid-to-soft tissue contrast, adding significant diagnostic value to the evaluation of a wide variety of spine disorders.

## Highlights

• Conventional spin echo imaging is limited by low spatial resolution and CSF pulsation artifact.
• CISS MRI enables submillimeter spatial resolution and myelographic contrast.
• Inherent flow compensation of the CISS technique reduces CSF pulsation artifact.
• CISS improves the delineation of a wide variety of spinal pathologies.

## 1 Introduction

High spatial resolution is a major challenge in magnetic resonance imaging (MRI) of the spine. In order to cover a large tissue volume, routine axial and sagittal spin echo (SE) sequences are typically 3–4 mm in thickness which can result in partial volume averaging and low spatial resolution. With significant improvements in imaging technology over the past few decades, MRI sequences utilizing steady-state free precession (SSFP) have become the staple of many clinical applications in neuroimaging. Such sequences have been shown to be particularly valuable for the evaluation of the cranial nerves [1], the cisternal spaces and inner ear [2], and the ventricular system [3]. For spine applications, balanced SSFP (b-SSFP) sequences such as constructive interference in steady-state (CISS) and fast imaging employing steady-state acquisition with phase cycling (FIESTa–c) offer the ability to image with submillimeter spatial resolution and high cerebrospinal fluid (CSF)-to-soft tissue contrast [4]. This allows excellent delineation of small caliber structures such as nerve roots, denticulate ligaments and adhesion bands, and allows discrimination of sub-millimeter thin walls of intraspinal cysts. This pictorial review highlights the role of CISS/FIESTA-C in providing detailed anatomic information on a wide range of complex spinal pathologies, illustrating the value of this supplemental high spatial resolution and high contrast resolution spine imaging.

## 2 Physics of CISS/FIESTA-C

In a gradient echo sequence, when the repetition time (TR) is kept shorter than the T2 relaxation of the tissue, residual transverse magnetization (TM) is left over after each radiofrequency (RF) pulse. In classic steady-state sequences, this residual transverse magnetization is not wasted (as in spoiled GRE sequences) but is instead fed back into longitudinal magnetization (LM) with the next RF pulse. After a train of RF pulses, the repeated flipping of longitudinal magnetization into transverse magnetization and vice versa results in a nonzero steady state for both LM and TM [5] [6].

**Fig. 1:**
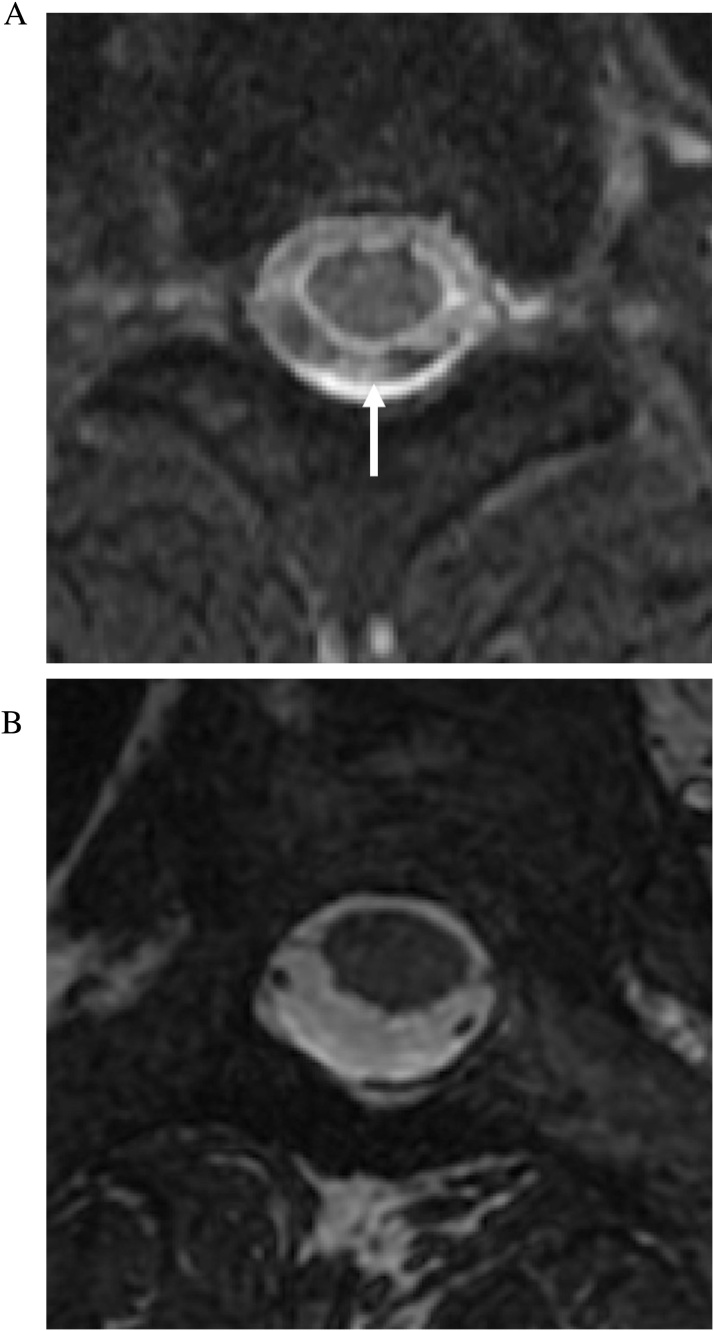
CSF pulsation artifact. (a) Axial T2-weighted FSE MRI image (slice thickness, 3 mm) of the upper cervical spine demonstrates CSF pulsation artifact within the thecal sac, more prominent at the dorsal aspect (arrow). (b) Axial FIESTA-C MRI image (slice thickness, 1.2 mm) through the same level demonstrates homogeneous high signal of CSF and elimination of the pulsation artifact.

For SSFP image formation, the two types of signals generated after each RF pulse include the free induction decay (FID) signal which has mixed T1 and T2* weighting and a spin echo from the prior RF pulse which has T2 weighting [5]. In a b-SSFP sequence, both the FID and the spin echo signals are used for image formation. Furthermore, the gradients applied in the slice selection, readout, and phase encoding directions are each balanced by gradients of opposite polarity so that there is no net dephasing of the transverse magnetization within each TR [5,6]. The gradient symmetry allows b-SSFP to have decreased sensitivity to motion and pulsation artifact (Fig. 1) and susceptibility compared to non-balanced SSFP sequences [5].

CISS (Siemens) and FIESTA-C (GE) are equivalent sequences which represent a further modification of the b-SSFP paradigm. These modifications help reduce phase shift errors that cause dark banding artifacts which may obscure the image. In these implementations, two successive three-dimensional (3D) b-SSFP sequences are acquired, one performed with RF pulses which have alternating +α and -α flip angles and the other with a constant +α angle. These two datasets will contain banding artifacts which are mutually shifted [5,6]. A maximum intensity projection of the two datasets is then created which minimizes the banding artifact in the final image and increases the signal-to-noise. The contrast of b-SSFP sequences such as CISS and FIESTA-C is a ratio of the T2/T1 of the tissues. Tissues with long T2 values, such as water and CSF, appear bright compared with solid tissues with lower T2. While fat has a shorter T2 value than fluid, its high T2/T1 ratio also results in a high signal on CISS/FIESTA-C [5,6].

Our patients were imaged on a 1.5 or 3.0 T GE scanner (GE Healthcare, Chicago, Ill) using a spine array surface coil. Multiplanar reformats were created on an independent PACS workstation. Scanning parameters for FIESTA-C are listed in Table 1.

**Table 1:** FIESTA-C MRI scanning parameters.

Sequence	FIESTA-C
Field Strength (T)	1.5 or 3.0
Imaging Plane	Sagittal
TE (ms)	Min 1.8
TR (ms)	4.6
Flip angle (degrees)	55
FOV (mm)	220
NEX	2
Matrix	256 × 256
Slice thickness (mm)	1.2
Voxel Size (mm)	0.86
Sections	68
Acquisition time (min)	2:48

Abbreviations: T, Tesla; TE, echo time; TR, repetition time; NEX, number of excitations.

## 3 Normal spinal anatomy

**Fig. 2:**
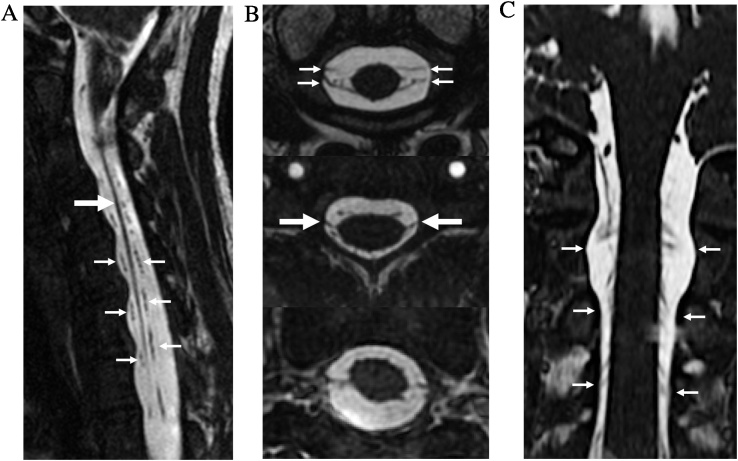
Normal Anatomy. Sagittal FIESTA-C MRI image (a) and axial and coronal reformatted images (b–c) demonstrate the detailed intradural anatomy of the cervical spine, including the denticulate ligament (large arrows in a–b), as well as the paired ventral and dorsal rootlets of the spinal nerves (small arrows in a–c).

**Fig. 3:**
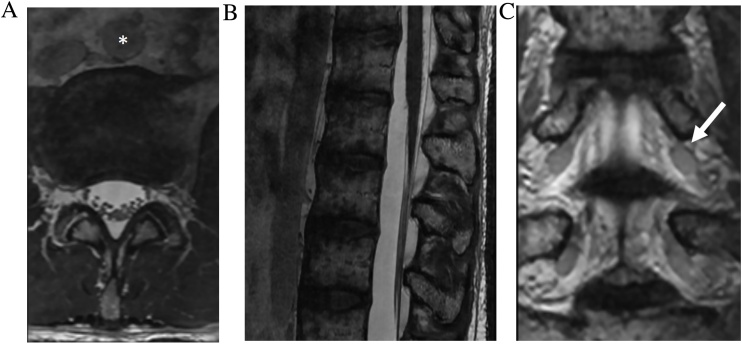
Normal anatomy. Axial, sagittal, and coronal FIESTA-C MRI images (a–c) demonstrates the high signal intensity of epidural fat and CSF, due to their similar high T2/T1 ratios. Solid structures such as disc annulus, cortical bone, muscles, ligaments, and neural structures have a similar low signal intensity. Blood within the major vessels have an intermediate signal intensity (asterisk). Dorsal root ganglia have an intermediate signal (arrow).

CISS MRI provides fine spatial resolution and high contrast between cerebrospinal fluid/fat and solid structures, allowing the delineation of fine anatomic details such as the ventral and dorsal rootlets of the spinal nerves and the denticulate ligaments (Fig. 2). The high signal intensities of fat and CSF are secondary to similar high T2/T1 ratios of these substances, whereas solid tissues tend to have low T2/T1 ratios and appear low in signal (Fig. 3) [7].

## 4 Degenerative discovertebral disease

**Fig. 4:**
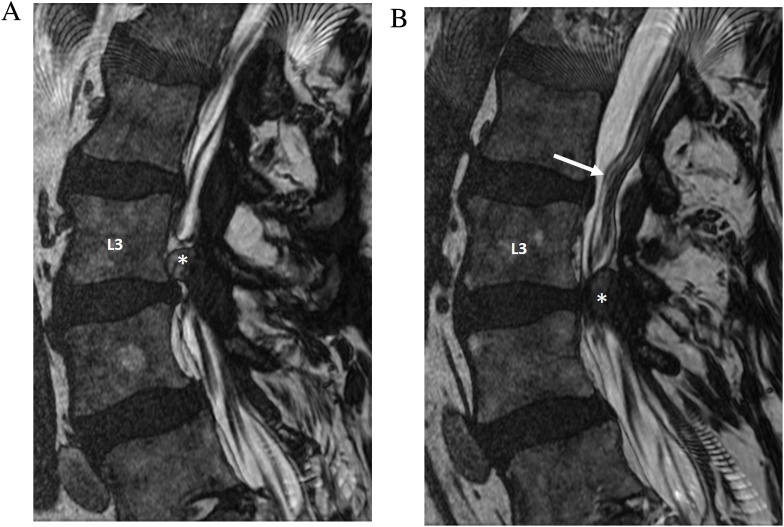
Spinal stenosis secondary to synovial cysts. A 75-year-old man who presented with radiculopathy. Sagittal FIESTA-C MRI images to the left (a) and to the right (b) of midline demonstrate bilateral synovial cysts (asterisks) which cause compression of the thecal sac and buckling of the cauda equina nerve roots (arrow).

MRI with conventional spin echo sequences is considered to be the modality of choice in the evaluation of degenerative spine disease with neurologic symptoms. However, MRI findings sometimes do not correlate with clinical symptoms which leads to diagnostic ambiguity. In particular, lateral recess nerve root compression has been reported to be underestimated by MRI in 28–29% of surgically confirmed cases, compared to 5–7% by conventional myelogram [8]. CT myelography has also been reported to be more sensitive for determining the levels for decompressive surgery compared to MRI with conventional sequences [9]. CISS may supplement the conventional MRI sequences by providing thinner sections, reduced partial volume effects, and greater myelographic contrast. In a study utilizing a CISS equivalent sequence in low field MRI, Maksymowicz et al [10] noted improved depiction of the boundaries of herniated discs and osteophytes with respect to the thecal sac and neural elements compared to T2-weighted FSE. While CISS is limited in the assessment of disc signal and differentiating disc elements, osteophyte, and ligamentous structures due to the similar signal intensity of these structures, the fine spatial resolution of CISS can clearly show the effect of degenerative disease on the thecal sac and nerve roots (Fig. 4).

## 5 Trauma and post-operative spine evaluation

**Fig. 5:**
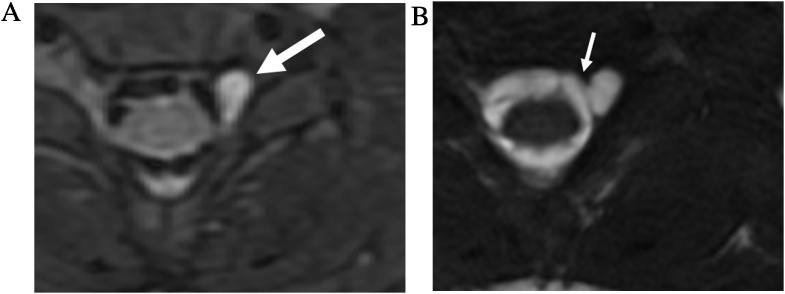
Brachial plexus birth injury. 6-month-old male with pseudomeningocele. Axial T2-weigted fat saturated FSE MRI image (a) demonstrates CSF intensity cyst in the left C6-7 neuroforamen. Intradural nerve roots are obscured by pulsation artifact. Axial FIESTA-C MRI image (b) shows the site of dural defect (thin arrow) and associated pseudomeningocele. The left sided intradural rootlets and exiting left C7 nerve root are absent, indicating avulsion.

**Fig. 6:**
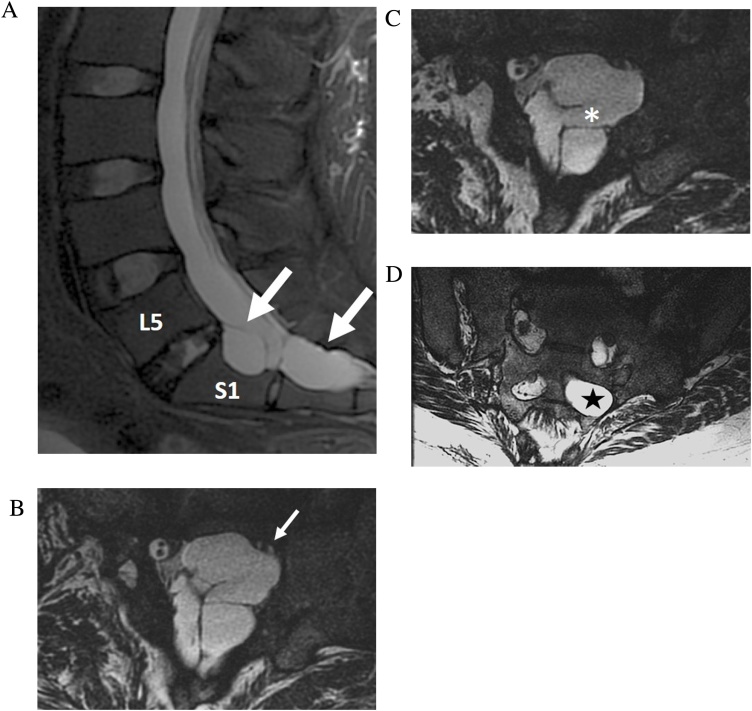
Post traumatic pseudomeningocele with nerve root avulsion. 25-year-old woman with chronic bowel and bladder incontinence and left leg pain after motor vehicle accident. Sagittal T2-weighted FSE MRI (a) shows a complex multiseptated post traumatic pseudomeningocele within the left S1 and S2 neuroforamen. Sequential axial FIESTA-C MRI images (b–d) resolve the site of dural tear (asterisk), the compressed left S1 nerve root within the S1 neuroforamen (small arrow), and absence of the left S2 nerve root (star) due to avulsion.

Traumatic pseudomeningocele are abnormal collections of CSF outside of the thecal sac formed secondary to nerve root avulsions. Its formation is suggestive of a preganglionic injury which portends a poor prognosis for recovery and may necessitate nerve transfer for partial functional recovery [11]. Small pseudomeningoceles and their causative dural defects can be very difficult on conventional T2-FSE images due to limited spatial resolution and pulsation artifacts. CISS can help resolve sites of dural tear (Fig. 6), and can demonstrate the integrity of intradural nerve roots with high sensitivity (89%), specificity (95%), and accuracy (92%) [12] without requiring contrast myelography. This may be beneficial particularly for neonates with birth related brachial plexus injury (Fig. 5).

**Fig. 7:**
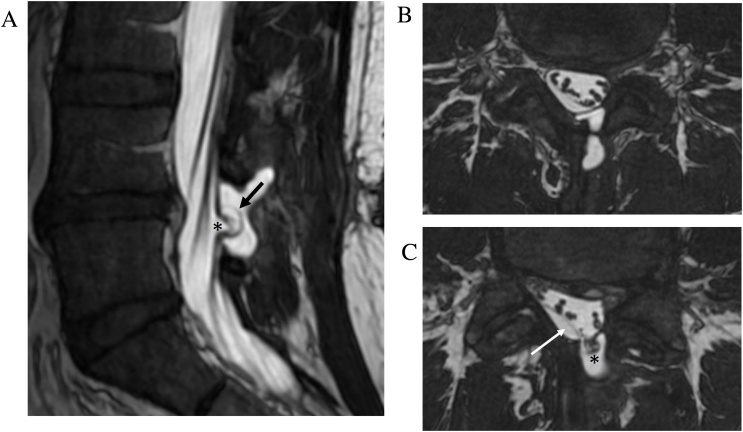
Post-operative pseudomeningocele with nerve root tethering. Sagittal FIESTA-C MRI image (a) shows a dural defect (arrow) and adjacent pseudomeningocele at the site of prior L4-5 laminectomy. Axial FIESTA-C MRI images (b–c) There is tethering and herniation of traversing sacral nerve roots (asterisk) through the dural defect. A subdural effusion with displacement of the arachnoid membrane (arrow) is also present.

Pseudomeningocele formation is a possible complication of intradural spinal surgery. Symptomatic pseudomeningoceles can lead to wound breakdown, nerve root and cord herniation, and intracranial hypotension [13]. Post-operative seroma is a common imaging mimic and the differentiation of these entities is important to direct neurosurgical management [14]. CISS can be useful to assess for dural defects and help differentiate seroma from pseudomeningocele (Fig. 7).

## 6 Vascular lesions

**Fig. 8:**
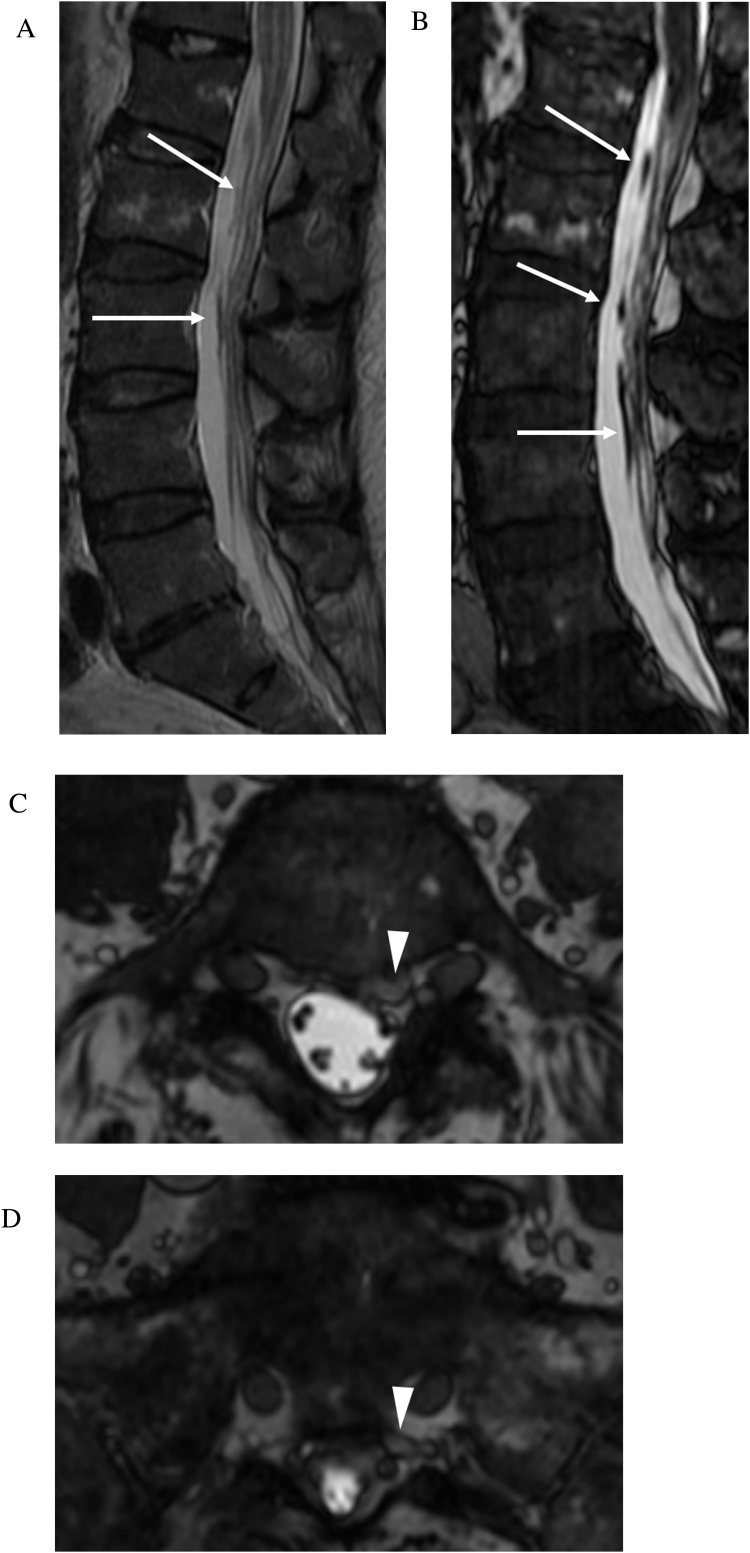

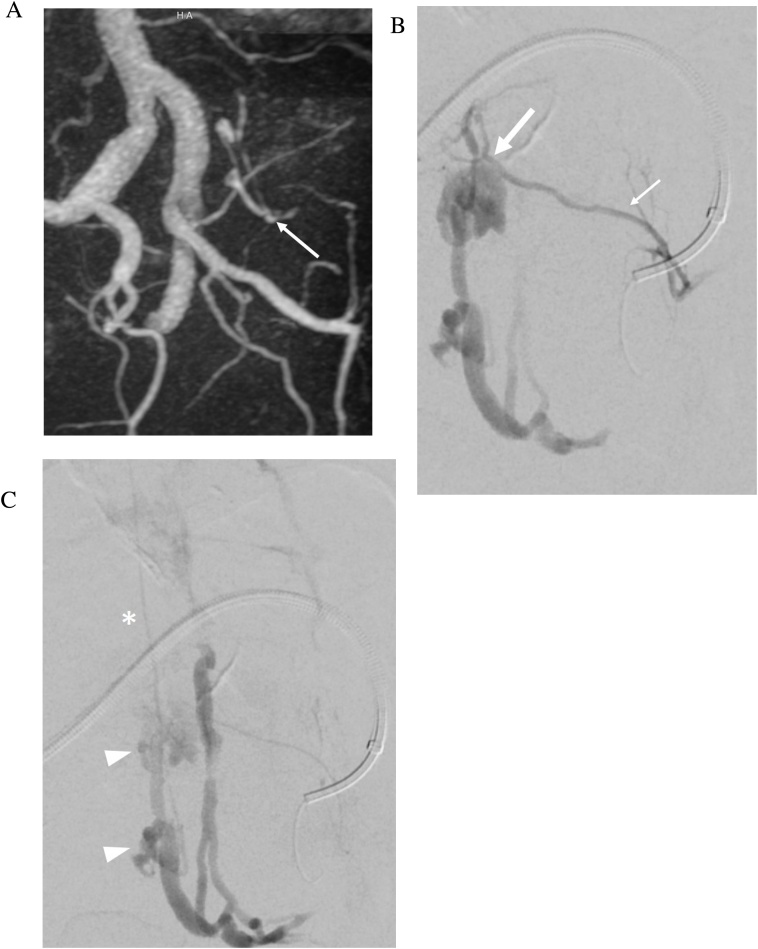
a Spinal dural arteriovenous fistula. 74-year-old man presenting with 5 months history of bilateral lower extremity weakness, numbness, and low back pain. Initial catheter spinal angiogram was negative for dural arterial venous fistula. Sagittal T2-weighted FSE MRI image (a) demonstrates central cord signal abnormality within the conus. Faint linear low signal is seen along the ventral aspect of the cauda equina (arrows). Sagittal FIESTA-C MRI image (b) clearly demonstrates a tortuous and enlarged filum terminale vein (arrows). Axial FIESTA-C MRI images (c–d) demonstrate tracking of the enlarged filum terminale vein into an engorged vessel within the left ventral epidural space at L5 and S1 (arrowheads). b Spinal dural arteriovenous fistula. 3D reconstruction of dynamic contrast enhanced MRA image (a) confirms a connection (arrow) between a branch of the left internal iliac artery (iliolumbar artery) and the abnormal vessel within the left L5 and S1 ventral epidural space. Digital subtraction angiogram images (b–c) shows selective contrast injection into the left iliolumbar artery (thin arrow) with demonstration of the fistula at the level of the L5-S1 neural foramen (thick arrow). Venous drainage is to dilated arterialized ventral epidural varices at L5 (arrowheads) from which an ascending filum vein is noted to fill (asterisk)..

Spinal dural arteriovenous fistula (dAVF) is an underdiagnosed entity which can lead to progressive myelopathy and morbidity if untreated [15]. CISS complements conventional FSE sequences in the evaluation of spinal dAVF and better demonstrates the associated tortuous and dilated venous structures [16]. This is particularly true of deep lumbosacral dAVFs that may lack the classic finding of prominent flow voids along the pial surface of the cord and are more difficult to diagnose compared with dAVFs at other locations. In concert with gadolinium enhanced dynamic MRA, CISS can help localize the fistula site and troubleshoot difficult cases which are initially negative on conventional digital subtraction angiography (Fig. 8a, b). CISS may identify the most probable site(s) of fistulous connection and reduce time, dose, and contrast usage during conventional angiography [17].

## 7 Arachnoiditis and arachnoid cysts

**Fig. 9:**
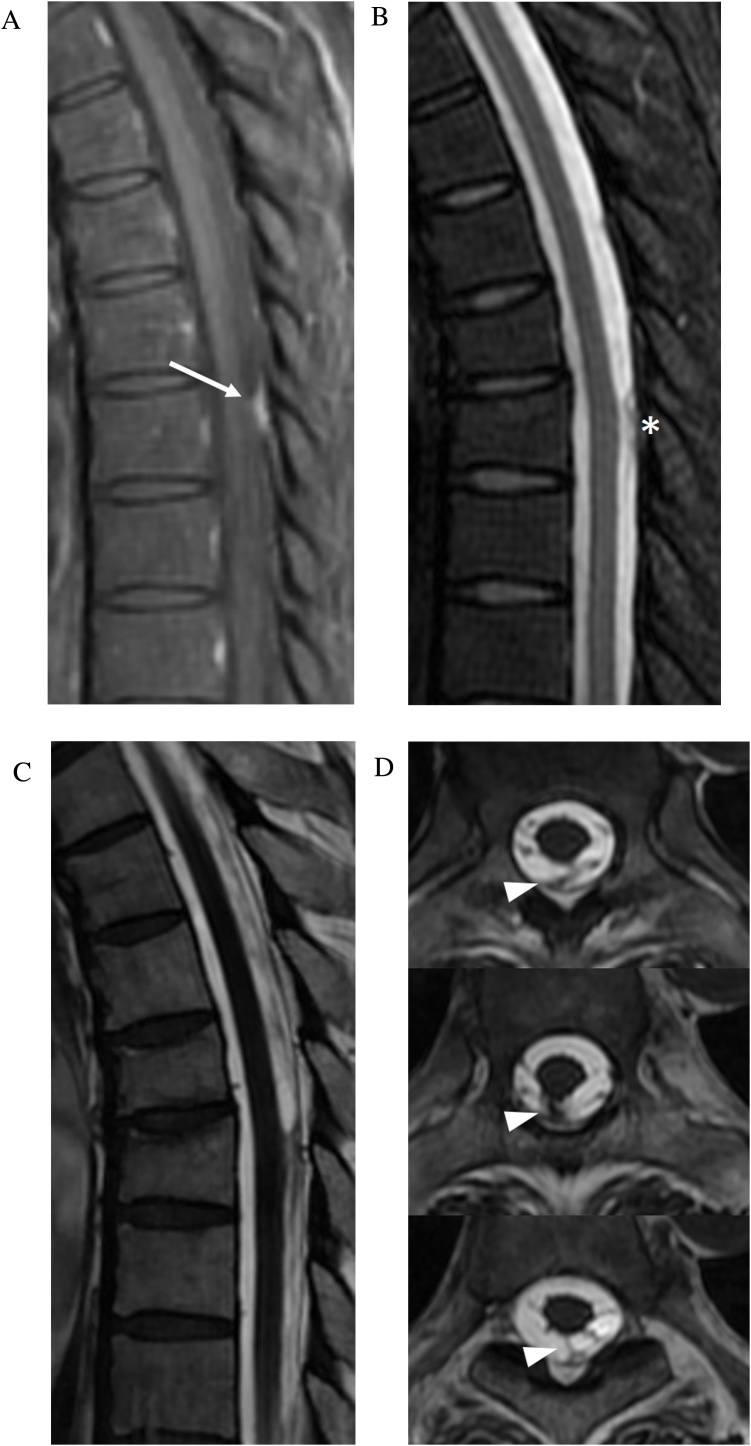
Arachnoid scar. 46-year-old man status post radiation therapy for nasopharyngeal carcinoma, complicated by intracranial abscesses. Sagittal contrast enhanced fat saturated T1-weighted FSE image (slice thickness, 3 mm) of the thoracic spine (a) demonstrates a focus of enhancement along the dorsal thecal sac (arrow), which could be interpreted as an metastasis. Sagittal T2-weighted fat saturated FSE image (slice thickness, 3 mm) (b) shows an irregular hypointense nodule (asterisk). Sagittal and axial FIESTA-C MRI images (c–f) resolves the lesion as irregular linear bands of low signal within the dorsal subarachnoid space (arrowheads) consistent with arachnoid scar.

Arachnoiditis refers to inflammation of the spinal roots, meninges and subarachnoid space. Post-inflammatory fibrinous exudates coat the arachnoid membrane and nerve roots, which adhere to each other and the dura, causing clumping and neurologic impairment. This may result from prior spinal hemorrhage, operation, trauma, infection, inflammation, and lumbar puncture. The high CSF-to-soft tissue contrast of CISS allows resolution of fine meningeal adhesions, nerve root displacement and clumping, and subarachnoid scarring. CISS delineates the level and extent of arachnoiditis and demarcates the site of adhesion which is important in planning surgical release [18,19]. CISS can also help differentiate between scarring and tumor in cases of abnormal enhancement (Fig. 9).

**Fig. 10:**
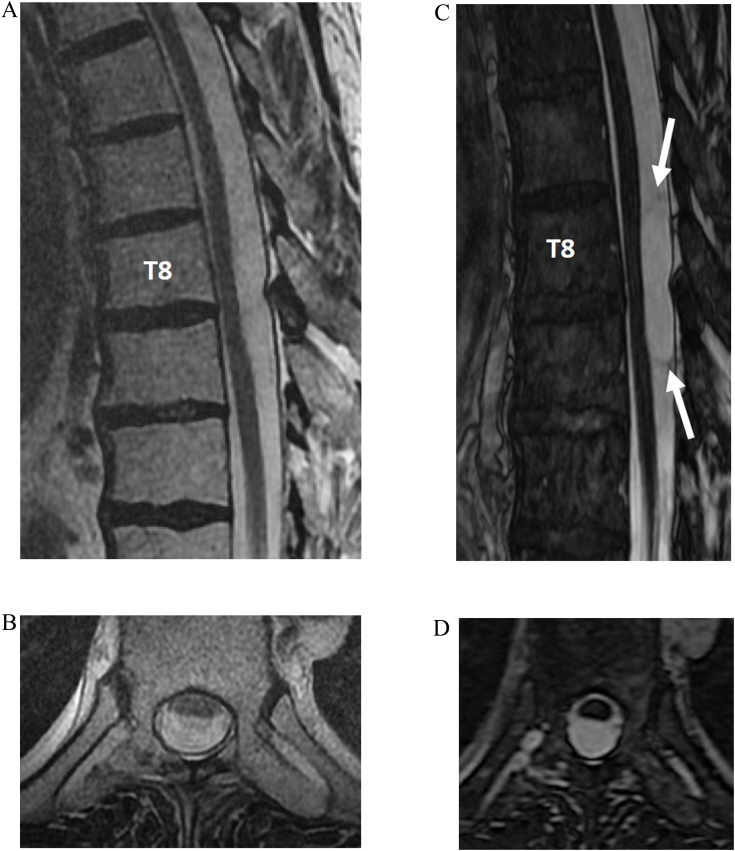
Intradural arachnoid cyst. Sagittal and axial T2-weighted MRI images (slice thickness, 3 mm) (a–b) demonstrate a subtle ventral displacement of the thoracic spinal cord. No definable lesions are visualized. Sagittal and axial FIESTA-C MRI images (c–d) demonstrate the delicate walls of the arachnoid cyst, as well as the homogenous signal intensity of the cyst contents.

Spinal cord compression secondary to arachnoid cysts is a rarely reported condition [20]. Intradural arachnoid cysts are CSF-filled sacs contained by arachnoid mater, with variable communication with the thecal sac. They are most commonly located in the dorsal subarachnoid space and may be congenital or acquired (post-trauma or inflammation, surgery, lumbar puncture, neoplastic, or parasitic lesions) [21]. Arachnoid cysts are difficult to detect on T2-weighted FSE images unless the cord is displaced. CISS/FIESTA-C can better define the contours of the cysts due to decreased pulsation artifacts, improved spatial resolution allowing depiction of a thin wall, and greater contrast between CSF and the cyst wall (Fig. 10).

## 8 Complex syringohydromyelia

**Fig. 11:**
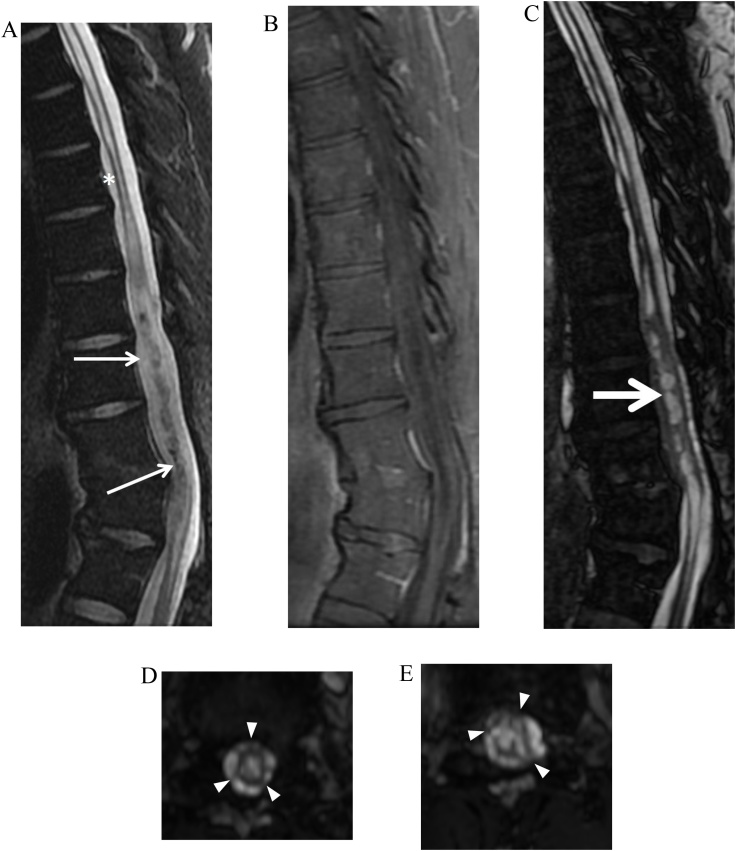
Arachnoid adhesions and complex syringohydromyelia. 34-year-old man with history of remote discitis osteomyelitis presents with worsening back pain, bilateral lower extremity weakness, and imbalance. Sagittal T2-weighted FSE MRI image (slice thickness, 3 mm) (a) demonstrates syringomyelia throughout the thoracic cord with myelomalacia cranially (asterisk) and relative expansion of the lower thoracic cord with central low signal (small arrows) which could be interpreted as a mass. Sagittal contrast enhanced T1-weighted FSE image (slice thickness, 3 mm) (b) demonstrates no abnormal enhancement. Sagittal (slice thickness, 1.2 mm) and axial FIESTA-C MRI images (c–e) clearly demonstrate the complex syrinx in the expanded lower cord with multiple internal septations (large arrow). Multifocal regions of arachnoid adhesion causing cord expansion are visualized (arrowheads).

Syringohydromyelia is defined as intramedullary cystic dilatation of the spinal cord with or without communication with the central canal. The underlying etiology may be related to CSF flow disturbance from injury (e.g. trauma or inflammation) or structural malformation (e.g. Chiari I or tethered cord syndromes) [22]. In many cases the underlying etiology is unknown. Although intrinsic cord signal intensity is best assessed on T2-weighted FSE sequences, CISS imaging offers more detailed delineation of intracavity septations and communications, as well as associated structural abnormalities such as tethered cord, arachnoiditis, and spinal dysraphism (Fig. 11). Detailed syrinx delineation is key in understanding of syrinx pathophysiology and helps plan surgical decompression [22].

## 9 Leptomeningeal metastases

**Fig. 12:**
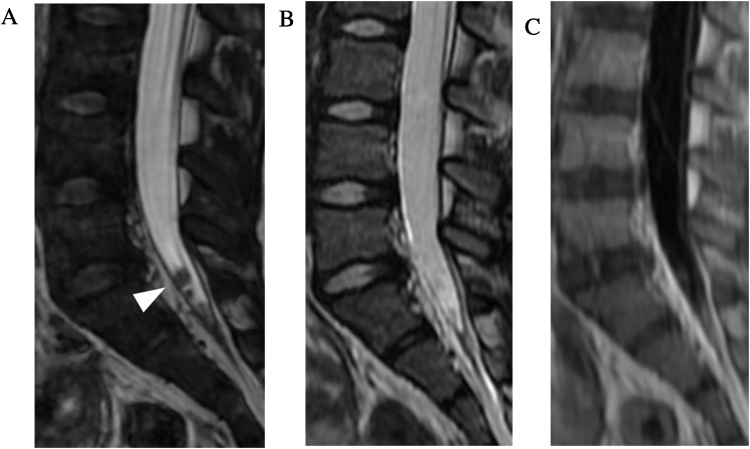
Leptomeningeal drop metastases. 7-year-old girl with history of diffuse intrinsic pontine glioma. Sagittal FIESTA-C MRI image (a) through the caudal thecal sac demonstrates nodular metastatic deposits (arrowhead), which are poorly seen on sagittal T2-weighted FSE (b) and sagittal contrast enhanced MRI images (c) (slice thickness, 3 mm).

Contrast enhanced MRI is the modality of choice in the imaging workup of spinal leptomeningeal metastases [23]. In the setting of primary brain tumors which may disseminate within the CSF such as medulloblastoma and pineoblastoma, accurate detection of drop metastases is particularly important as it affects the treatment planning for radiation and chemotherapy [24]. While conventional spin echo sequences can demonstrate drop metastases, evaluation can be limited due to pulsation artifacts and volume averaging. CISS allows for more sensitive evaluation of subtle findings of nodularity or nerve root thickening which can raise concern for drop metastases (Fig. 12) [25].

## 10 Spinal dysraphisms

**Fig. 13:**
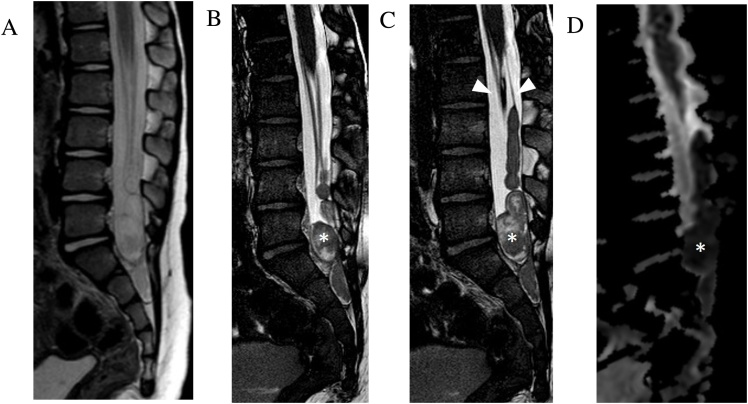
3-month-old male with congenital spinal dysraphism. Sagittal T2-weighted FSE (a), FIESTA-C (b–c) and ADC (d) demonstrate thickened tethered bands (arrowheads) and multiple masses (asterisks) in the caudal thecal sac which are better seen on FIESTA-C images. ADC image demonstrates reduced values within the masses compatible with epidermoid cysts.

**Fig. 14:**
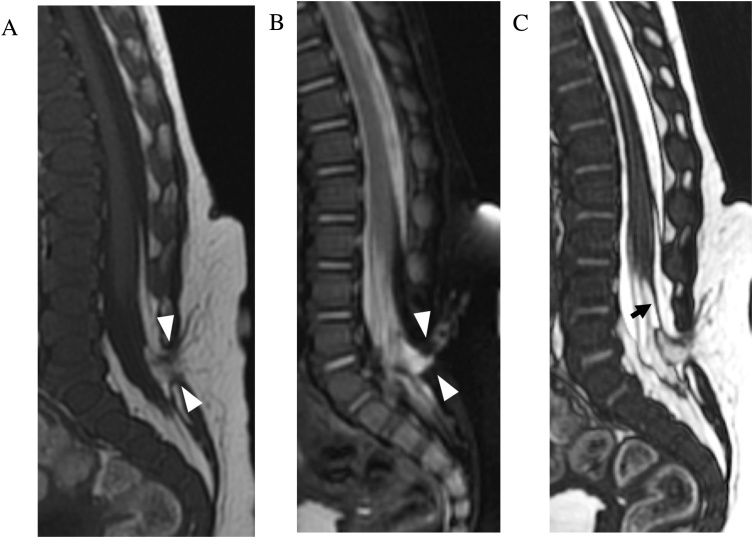
Dorsal epidural lipoma with tethered cord. 3-month-old male with palpable subcutaneous mass over the sacrum on physical exam with no cutaneous discoloration. Sagittal T1-weighted FSE (a), T2-weighted FSE (b) and FIESTA-C (c) MRI images demonstrate a spina bifida defect at L5-S1. A lumbosacral subcutaneous lipoma extends through the spina bifida defect (arrowheads) into the dorsal epidural space. Compared to T2-weighted FSE, FIESTA-C better demonstrates the filum terminale (arrow) which is tethered to the dorsal epidural lipoma and the low-lying conus.

Pediatric spinal dysraphism encompasses a wide spectrum of congenital anomalies resulting from abnormal neural tube closure and are divided into two main categories of open and closed defects [[26], [27], [28]]. MRI is the study of choice in the evaluation of spinal dysraphism, providing superior soft tissue contrast for diagnosis and surgical planning [29]. While conventional multiplanar spin echo sequences are able to define the gross morphology of lesions in spinal dysraphism, CISS is superior in depicting the fine details of nerve position within dural ectasias and lateral meningoceles. Its high CSF-to-nerve contrast and spatial resolution allows for clear definition of nerve relationship to thickened filum terminale, overlying lipomas, dermal sinus tracts, and intradural masses (Fig. 13, Fig. 14) [30,31]. The delineation of precise anatomy provides useful information for surgeons prior to spinal dysraphism repair [32]. Post-operative complications of repaired myelomeningocoele are also better demonstrated on CISS, including dural tear, nerve tethering, cord herniation, and cord cavitation.

## 11 Limitations

While CISS utilizes consecutive runs of 3D b-SSFP to minimize banding artifacts, these can still occur in areas of field inhomogeneity such as air-bone and soft tissue interfaces. These artifacts can be more pronounced at higher magnetic field strength (3.0 T compared to 1.5 T) secondary to increased sensitivity to susceptibility [33]. Despite the inherent flow compensation of CISS due to gradient symmetry, turbulent and rapid flow can still result in phase dispersion and signal loss [5].

While CISS accentuates the contrast between CSF and solid tissues, the intrinsic contrast resolution of neural structures is poor compared to spin-echo images. Furthermore, in cases such as collapsed myelomeningocele or severe spinal stenosis where neural structures are no longer surrounded by high signal CSF, CISS is unable to provide any more useful information than conventional spin-echo sequences. Therefore, CISS must be interpreted in combination with conventional T1- and T2-weighted images and is best used as a supplemental problem-solving sequence. This added acquisition time can be problematic for patients who are unable to tolerate the increased scan time, increasing the likelihood of motion artifact and incomplete exams. However, acquisition time can be improved by scanning in the sagittal plane along the short axis of the spine and reformatting in the remaining planes.

## 12 Conclusion

CISS is a useful troubleshooting sequence which provides the fine spatial resolution lacking in conventional spin echo sequences and accentuated contrast between CSF and neural structures. When used in concert with routine sequences, CISS can provide significant added value in a wide variety of spine applications.
